# Profiling and quantitative analysis of underivatized fatty acids in *Chlorella vulgaris* microalgae by liquid chromatography‐high resolution mass spectrometry

**DOI:** 10.1002/jssc.202100306

**Published:** 2021-06-24

**Authors:** Carmela Maria Montone, Sara Elsa Aita, Martina Catani, Chiara Cavaliere, Andrea Cerrato, Susy Piovesana, Aldo Laganà, Anna Laura Capriotti

**Affiliations:** ^1^ Department of Chemistry Sapienza University of Rome Rome Italy; ^2^ Department of Chemistry and Pharmaceutical Sciences University of Ferrara Ferrara Italy; ^3^ CNR NANOTEC Campus Ecotekne University of Salento Lecce Italy

**Keywords:** biofuel, compound discoverer, fatty acids, lipidomics, untargeted analysis

## Abstract

*Chlorella vulgaris* is a popular microalga used for biofuel production; nevertheless, it possesses a strong cell wall that hinders the extraction of molecules, especially lipids within the cell wall. For tackling this issue, we developed an efficient and cost‐effective method for optimal lipid extraction. Microlaga cell disruption by acid hydrolysis was investigated comparing different temperatures and reaction times; after hydrolysis, lipids were extracted with *n*‐hexane. The best recoveries were obtained at 140°C for 90 min. The microalgae were then analyzed by an untargeted approach based on liquid chromatography with high‐resolution mass spectrometry, providing the tentative identification of 28 fatty acids. First, a relative quantification on the untargeted data was performed using peak area as a surrogate of analyte abundance. Then, a targeted quantitative method was validated for the tentatively identified fatty acids, in terms of recovery (78‐100%), intra‐ and interday relative standard deviations (<10 and <9%, respectively) and linearity (*R* ^2 ^> 0.98). The most abundant fatty acids were palmitic, palmitoleic, oleic, linoleic, linolenic, and stearic acids.

**Article Related Abbreviations**
AGC: automatic gain control
DAG: diacylglycerol
DDA: data‐dependent acquisition
FA: fatty acid
FWHM: full width at half maximum
*i*‐PrOH: isopropanol
MeOH: methanol
MRM: multiple reaction monitoring
MUFA: monounsaturated fatty acid
PUFA: polyunsaturated fatty acid
RT: room temperature
SFA: saturated fatty acid
TAG: triacylglycerol

## 1 INTRODUCTION

Microalgae are a rich source of precious bioactive compounds, such as lipids [1, 2, 3, 4], carotenoids [5], phenolic compounds [6], bioactive peptides, and amino acids [7, 8, 9]; for this reason, they found application in the pharmaceutical and nutraceutical fields as valuable sources of new food and functional products [10, 11, 12]. Fatty acids (FAs) are among the major constituents of microalgal biomass and typically range between 5 and 50% of the cell dry weight [13, 14]. They are mainly present as glycerolipids, primarily consisting of phospholipids and glycolipids, which have an essential role in cell structure, and triacylglycerols (TAGs), primarily responsible for energy storage [15]. In particular, TAGs possess a broad spectrum of commercial applications, such as the production of biofuels [3, 16, 17], bulk chemicals [18], nutraceuticals, especially for the production of omega‐3 FAs [19, 20], and food commodities [13]. Microalgae mainly produce FAs with chain lengths of 16 and 18 carbon atoms, but some species can synthesize FAs of up to 24 carbon atoms in length. The presence or absence of saturation plays an important role in biofuel properties; for example, saturated FAs (SFAs) provide good oxidative stability and ignition properties, while polyunsaturated fatty acids (PUFAs) have good cold‐flow characteristics [21].

*Chlorella vulgaris* is currently the second most commercially produced microalga due to its relative ease of cultivation. However, this microalgal strain's industrial productivity is hampered by its strong cell wall, making the extraction of functional molecules an issue [22]. Evaluating cost‐effective cell disruption methods to maximize lipid extraction from microalgae is crucial for identifying promising biofuel‐producing species. The appropriate method's choice depends on the microalgae species and cell wall characteristics [23, 24]. Several cell‐disruption methods, such as mechanical, biological, and chemical ones, have been used to develop efficient downstream processes to recover intracellular lipids, mostly free FAs, and pigment components from *C. vulgaris* [25]. Mechanical methods require energy inputs in the forms of shear forces, electrical pulses, waves, and heat; the main employed approaches are bead milling, high‐pressure homogenization, hydrodynamic cavitation, and ultrasonication [26]. It has recently been demonstrated that the combination of two mechanical methods, such as sonication‐assisted high‐speed homogenization, was more efficient in lipid extraction than the single use of sonication or homogenization. Moreover, a chloroform/MeOH mixture gave a higher lipid yield than *n*‐hexane, with 238 and 152 mg lipid/g cell, respectively [24].

Physical approaches are not suitable for large‐scale production, since they are expensive and could cause final product degradation [25, 27]. Biological techniques are based on the use of lysis enzymes or algicidal treatment. These methods have significant advantages, such as their biological specificity, mild operating conditions, and low energy consumption; however, they have not been applied at a large scale because of their low cost efficiency [28]. Chen et al. [29] demonstrated that the use of *Flammeovirga yaeyamensis*'s enzyme led to an increase of about 63% of lipid recovery in *C. vulgaris* compared to commercial amylase and cellulose enzymes. Nevertheless, the enzyme cost usually is higher than that of chemical and physical cell‐disruption methods, and in any case, the cell wall disruption rate is lower [25, 26].

Chemical methods, based on the use of strong acids, are nowadays the best solution in terms of high extraction efficiency and low cost for microalgae cell wall disruption compared to other chemical agents such as solvents, salts, and surfactants. In particular, 1% of sulfuric acid showed a lipid extraction yield of 935 mg/g from wet *C. vulgaris* [30]. Given the above, in this work, acid hydrolysis followed by solvent extraction was performed and optimized to weaken the microalgal cell walls of *C. vulgaris*. The effect of acid hydrolysis on FAs productivity was also investigated. An untargeted ultra (U)HPLC‐high resolution (HR) MS/MS approach without chemical derivatization was applied to characterize lipid extracts. Identification of FAs was performed by Compound Discoverer software using a predefined workflow for food analysis. Finally, the method was validated for the quantitative analysis of the 28 tentatively identified FAs from the untargeted analysis.

## 2 MATERIALS AND METHODS

### 2.1 Chemicals and reagents

HPLC‐grade chloroform, MeOH, and water used for sample pretreatment were supplied by VWR International (Milan, Italy). Ultrapure water and isopropanol (*i*‐PrOH) of LC‐MS grade were purchased from Thermo Fisher Scientific (Waltham, MA, USA); LC‐MS grade MeOH was provided by Romil Pure Chemistry (Pozzuoli, NA, Italy).

Ammonium acetate, acetic acid, sulfuric acid, and *n*‐hexane were purchased from Merck (St. Louis, MO, USA). The 35 lipid standards constituted of 28 FAs, 1 TAG, 1 diacylglycerol (DAG), and 5 phospholipids are reported in Supporting information Table S1 with all valuable information.

### 2.2 Preparation of stock solutions, working standard solutions, and calibration mixtures

Standard and stock solution preparation was always performed using glass equipment to avoid lipid adsorption to plastics, and tubes were covered with aluminum foil to prevent lipid oxidation. Stock (10 mg/mL) and working solutions (1 mg/mL) were prepared in MeOH for each compound and stored at −20°C. The quantitative analysis was carried out for all identified FAs. Eight‐point calibration curves were constructed in the range of 0.25‐47.00 μg/mL for all standards. Quantification was performed by matrix‐matched calibration.

### 2.3 Microalgal strain and growth medium

*Chlorella vulgaris* was isolated and maintained in BG11 medium with the addition of 0.25% of glucose under constant illumination at 1000 uE at room temperature (RT, 25°C), aerated (3 L/min), and maintained under continuous agitation with a magnetic stirrer. Illumination was provided 24 h/24 by cool light fluorescent lamps, positioned at a 10 cm distance from the bottle surface. Biomass concentration was determined as dry weight by filtering 10 mL of the suspension on glass microfiber filters (0.7 μm) previously dried at 105°C. The filters were again dried after filtration at 105°C until reaching constant weight. The algal biomass was decanted overnight; then, the supernatant was discarded, and the solid residue was harvested via centrifugation at 25°C for 10 min at 3000× *g*. Pellets were lyophilized and then ground to a fine powder with liquid nitrogen.

The sample was stored at 20°C until analysis.

### 2.4 Acid hydrolysis of *Chlorella vulgaris* and extraction of fatty acids

Acidic hydrolysis was performed on 0.5 g of microalgal biomass in 5 mL of H_2_SO_4_ at 5%v/v; hydrolysis was carried out in autoclave (Autoclave vapour‐lineeco, VWR), testing and comparing three temperatures (RT, 70°C, and 140°C) for three different reaction times (60 min, 90 min, and overnight). For each condition, the acid‐hydrolyzed mixtures were extracted twice with 3 mL of *n*‐hexane under vortex agitation for 30 min. The effects of hydrolysis temperature and reaction time were evaluated by spiking the dried microalgae (0.5 mg) with a mixture of standard lipids at 0.5 mg/mL. The following standard lipids were used: 15:0‐18:1‐15:0 TAG, 15:0‐18:1 DAG, 14:0 PA, 14:0 phosphatidylglycerol, 14:0 phosphatidylcholine, 14:0 phosphatidylethanolamine, 14:0 phosphatidylserine. The sample was spiked either before (*A*_set1_) or after the hydrolysis and extraction process (*A*_set2_) to calculate the hydrolysis percentage, according to the following equation:

(1) $$\%\;hydrolysis=100-\left(\frac{A_{set1}}{A_{set2}}\times \;100\right)$$

where *A*_set1_ is the analyte area in the sample fortified before extraction and *A*_set2_ is the analyte area in the spiked extract.

The hexane extracts were pooled and evaporated to dryness in an IKA RV 8 rotary evaporator (IKA‐Werke GmbH & Co. KG, Staufen, Germany). The residue was redissolved in 200 μL of MeOH/H_2_O/CHCl_3_, 80:15:5 (v/v/v) 5 mmol/L H_3_PO_4_. The best solubilization of FAs was achieved by adding solvents in the following order: CHCl_3_, MeOH, and H_2_O. The sample was stored at −20°C and diluted five times, with the same solvent mixture, before analysis.

### 2.5 Ultra‐high‐performance liquid chromatography‐high‐resolution mass spectrometry analysis

The UHPLC Vanquish binary pump H (Thermo Fisher Scientific, Bremen, Germany), equipped with a thermostated autosampler and a thermostated column compartment, was used to analyze FAs in microalgae samples. Twenty microliter of each sample was injected on a Hypersil Gold (50 × 2.1 mm i.d., 1.9 μm particle size) equipped with a Security guard Hypersil Gold (4 × 2.1 mm i.d., 5 μm particle size), both from Thermo Fisher Scientific. The column was operated at 300 μL/min at 40°C. Mobile phases were H_2_O/MeOH 70:30 (v/v) (A) and MeOH/*i*‐PrOH 60:40 (v/v) (B), both containing 0.05% acetic acid and 5 mmol/L ammonium acetate. The gradient was as follows: 20% B for 2 min; 20‐99% B in 18 min; 99% B for 15 min; the column was finally re‐equilibrated at 20% B for 9 min. The chromatographic system was coupled to a Q Exactive mass spectrometer (Thermo Fisher Scientific, Bremen, Germany) via an ESI source. The ESI source was operated in the negative polarity ionization mode with the following parameters: spray voltage 2500 V; capillary temperature 320°C; auxiliary gas at 15 arbitrary units (a.u.); auxiliary gas heater temperature 400°C; sheath gas 35 a.u.; S‐lens RF level was 100%. HRMS analysis was performed in the range *m*/*z* 200‐2000 with a resolution (full width at half maximum, FWHM, at *m*/*z* 200) of 70 000. The automatic gain control (AGC) target value was 5 × 10^5^, with a max ion injection time set of 200 ms. Top five data‐dependent acquisition (DDA) was used, with 35 000 resolution (FWHM at *m*/*z* 200) for MS/MS analysis. Higher‐energy collisional dissociation was performed at 40% normalized collision energy, using an isolation window width of 2 *m*/*z* and AGC target value of 5 × 10^5^. Dynamic exclusion was set to 6 s. Raw data files were acquired by Xcalibur software (version 3.1, Thermo Fisher Scientific). Three technical replicates were performed for each experimental replicate. Runs were performed on the same day.

### 2.6 Data analysis and fatty acid identification

Compound identifications were obtained according to level 2a confidence level in metabolomics analysis [31]. FA identification was carried out by Compound Discoverer™ 3.1 (Thermo Fisher Scientific) using the Food Research workflow template with few modifications. After spectra selection, alignment, and compound detections, adducts were grouped, and blank signals were removed. The *Fill Gaps* tool enabled a better evaluation of peak areas. Spectra matching was performed against FooDB and Lipid Maps databases with a mass tolerance of 5 ppm. The *Apply Spectral Distance* tool, which provides a ranking for compound annotation based on isotopic pattern comparison, was enabled, and the *Apply mzLogic* tool was used to rank annotations for unknown compounds based on MS/MS data.

### 2.7 Method validation for fatty acids in *Chlorella vulgaris*

The analytical method validation was carried out following the Food and Drug Administration document for bioanalytical method validation guidance for industry (https://www.fda.gov/regulatory‐information/search‐fda‐guidance‐documents/bioanalytical‐method‐validation‐guidance‐industry). Analyte stability was initially checked as freeze and thaw stability test, short‐term temperature stability test, long‐term stability test, stock solution stability test, and postpreparative stability test.

The method validation was carried out using selectivity, precision, recovery, and the analyte calibration curves in spiked samples. Compound identification was accepted if the retention time, accurate mass, and MS/MS fragmentation of the candidate compound in the sample matched the reference standard ones. The nonspiked microalgae samples were used to evaluate the absence of coeluted interferences at the same analytes retention time. Accuracy, precision, and recovery were determined by spiking microalgae samples before extraction and hydrolysis at three different concentration levels (C_1_ 0.02 μg/mL; C_2_ 0.50 μg/mL; C_3_ 1.00 μg/mL) with the 28 FAs reported in Supporting information Table S1. Recoveries (*R*, %) were calculated using the following equation:

(2) $$R\%=\frac{A_{set1}-A_{0}}{A_{set2}}\times 100$$

where *A_0_* is the compound area in the sample without any spiking (endogenous amount).

Precision was assessed as intraday precision (repeatability) and interday precision (intermediate precision), and the values were expressed as RSD. Signal suppression or enhancement due to matrix effect (ME, %) was evaluated as follows:

(3) $$\mathrm{ME}\%=\frac{A_{set2}-A_{0}}{A_{set3}}\;\;100$$

where *A*_set3_ is the standard area in pure solvent.

## 3 RESULTS AND DISCUSSION

### 3.1 Optimization of hydrolysis conditions

Acid hydrolysis with diluted sulfuric acid represents one of the best choices in industrial applications, especially for the low costs and ease of application. Moreover, compared with other strong acids, sulfuric acid provides a higher hydrolysis yield [32]. Although acid hydrolysis is a widely used method, the optimization of conditions is closely dependent on microalgae species and specific growth conditions. Three hydrolysis temperatures (RT, 70, and 140°C) and three reactions time (60 min, 90 min, and overnight) were evaluated and compared. The sulfuric acid concentration was not optimized, since several papers highlighted that 5% was the optimal choice, leading to an increased lipid yield compared to a more diluted acid [33]. On the other hand, Takisawa et al. [34] demonstrated that a higher concentration of sulfuric acid could degrade FAs.

**FIGURE 1:**
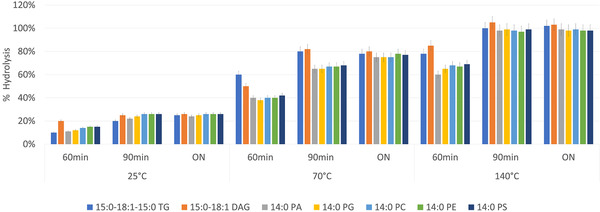
Effect of temperature and reaction time on the hydrolysis percentage of *C. vulgaris* cell walls using 5% of sulfuric acid. Percentages are reported for the seven standard lipids

The effects of temperature and reaction time on the hydrolysis percentage are shown in Figure 1 and were calculated following the Eq. 1. The maximum hydrolysis percentage (97‐100%) was obtained at 140°C after 90 min and overnight. Ninety minutes was selected as the best reaction time since it is preferred to carry out reactions at high temperatures for the shortest possible time to avoid degradation. The hydrolysis percentages at 25 and 70°C were in the range of 10‐26 and 38‐82%, respectively, for all the three tested reaction times. All hydrolysis percentages for every standard lipid, at every temperature and every reaction time, are reported in detail in Supporting information Table S2.

### 3.2 Fatty acid profiling in *Chlorella vulgaris* microalgae

Untargeted characterization of hydrolyzed FAs was carried out by UHPLC‐HRMS/MS. Before injection onto the UHPLC column, different solvent mixtures were tested to reach the best ionization rate and solubilization of the analyzed compounds in the extract resuspension mixture. Indeed, the solubility of FAs is a crucial factor in their identification. MeOH/H_2_O/CHCl_3_ (80:15:5, v/v/v) with 5 mmol/L H_3_PO_4_ was chosen as the best mixture for analyte injection; this mixture allowed obtaining a four‐time higher ionization efficiency. Furthermore, it has been proven that a small quantity of phosphoric acid into the resolubilization mixture can reduce peak tailing [35].

**FIGURE 2:**
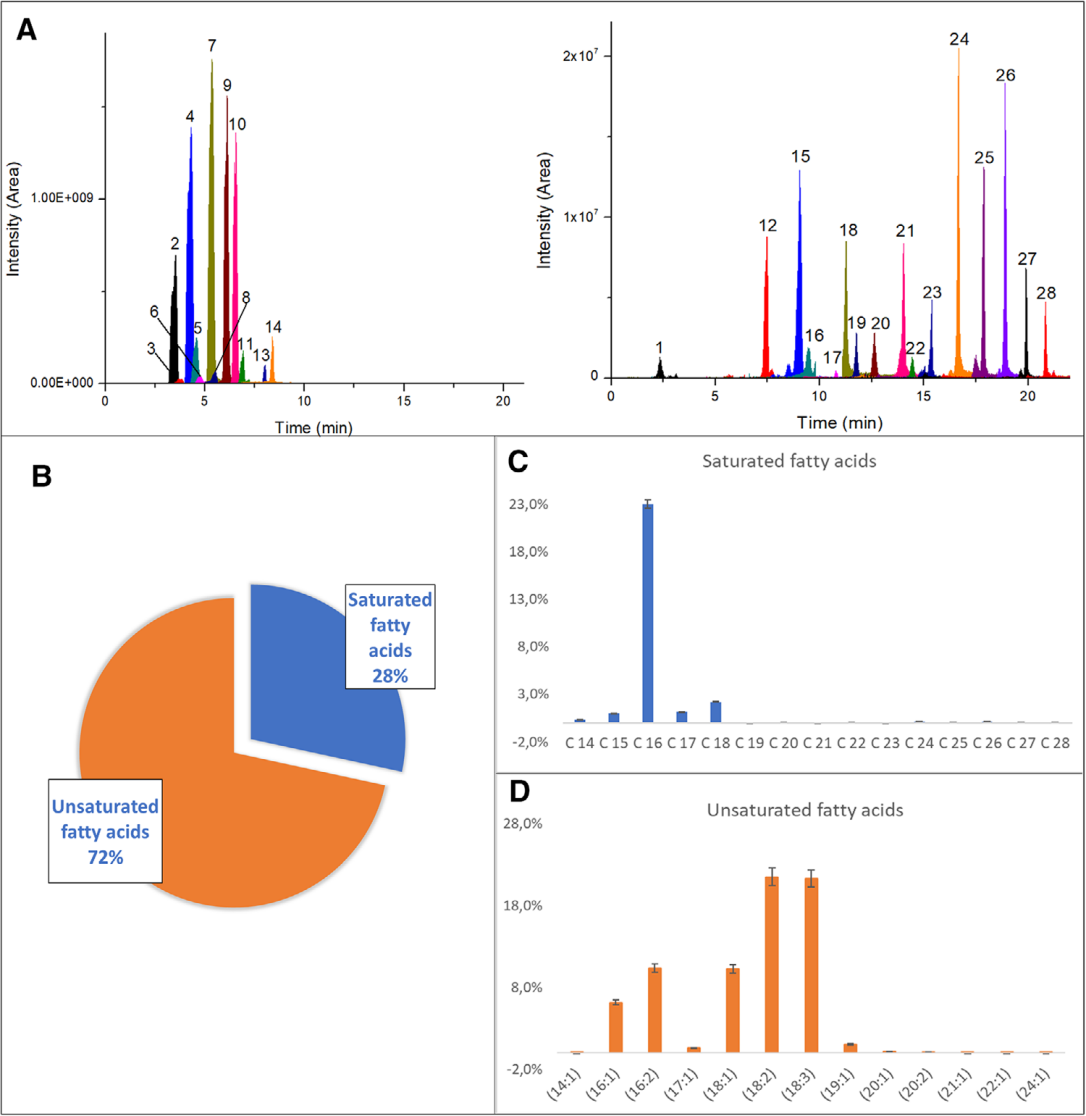
(A) Total ion chromatogram of FA species, with an area >2.5 × 10^8^ (left) and <2.5 × 10^8^ (right), extracted from *C. vulgaris* and identified by the untargeted analysis (compounds are marked by the same numbers as reported in Table 1); (B) pie chart showing the percentages of tentatively identified FAs according to their degree of unsaturation; (C) percentage distribution of individual saturated FAs relative to the total saturated FA content; (D) percentage distribution of individual unsaturated FAs relative to the total unsaturated FA content

Figure 2A shows the chromatographic separation of the 28 tentatively identified FAs. These identified FAs comprised 15 SFAs, 8 monounsaturated FAs (MUFAs), and 4 PUFAs, and they are reported in Table 1. Details on adduct, molecular weight, *Δ* error in ppm, major product ions, and peak area of tentatively identified FAs are reported in Supporting information Table S3.

**TABLE 1:** Tentatively identified FAs in *C. vulgaris* sample

Compound number*^a^*	Name	*t_R_* (min)	Molecular formula	Adduct	Exact mass (*u*)	Δ mass (ppm)	Relative abundance (%)
1	Myristoleic acid (14:1)	2.8	C_14_H_26_O_2_	[M‐H]^−^	226.1929	−1.8	<0.1
2	Hexadecadienoic acid (16:2)	3.5	C_16_H_28_O_2_	[M‐H]^−^	252.2087	−0.9	10.3
3	Myristic acid (14:0)	3.9	C_14_H_28_O_2_	[M‐H]^−^	228.2086	−1.4	0.3
4	Linolenic acid (18:3)	4.3	C_18_H_30_O_2_	[M‐H]^−^	278.2240	−2.3	21.3
5	Palmitoleic acid (16:1)	4.6	C_16_H_30_O_2_	[M‐H]^−^	254.2243	−1.0	6.2
6	Pentadecanoic acid (15:0)	5.0	C_15_H_30_O_2_	[M‐H]^−^	242.2244	−0.9	1.0
7	Linoleic acid (18:2)	5.3	C_18_H_32_O_2_	[M‐H]^−^	280.2397	−2.0	21.5
8	Heptadecenoic acid (17:1)	5.8	C_17_H_32_O_2_	[M‐H]^−^	268.2492	−2.2	0.6
9	Palmitic acid (16:0)	6.1	C_16_H_32_O_2_	[M‐H]^−^	256.2396	−2.4	23.0
10	Oleic acid (18:1)	6.6	C_18_H_34_O_2_	[M‐H]^−^	282.2553	−2.0	10.3
11	Margaric acid (17:0)	7.3	C_17_H_34_O_2_	[M‐H]^−^	270.2556	−0.9	1.1
12	Eicosadienoic acid (20:2)	7.6	C_20_H_36_O_2_	[M‐H]^−^	308.2713	−0.7	0.1
13	Nonadecenoic acid (19:1)	8.2	C_19_H_36_O_2_	[M‐H]^−^	296.2707	−1.0	1.0
14	Stearic acid (18:0)	8.6	C_18_H_36_O_2_	[M‐H]^−^	284.2711	−1.4	2.2
15	Gadoleic acid (20:1)	9.2	C_20_H_38_O_2_	[M‐H]^−^	310.2869	−0.8	0.2
16	Nonadecanoic acid (19:0)	9.7	C_19_H_38_O_2_	[M‐H]^−^	298.2869	−1.0	<0.1
17	Heneicosenoic acid (21:1)	10.1	C_21_H_40_O_2_	[M‐H]^−^	324.3025	−1.0	<0.1
18	Arachidic acid (20:0)	11.4	C_20_H_40_O_2_	[M‐H]^−^	312.3025	−1.0	0.1
19	Brassidic acid (22:1)	11.9	C_22_H_42_O_2_	[M‐H]^−^	338.3182	−0.7	<0.1
20	Heneicosanoic acid (21:0)	12.9	C_21_H_42_O_2_	[M‐H]^−^	326.3181	−1.1	<0.1
21	Behenic acid (22:0)	14.2	C_22_H_44_O_2_	[M‐H]^−^	340.3336	−1.7	0.1
22	Nervonic acid (24:1)	14.7	C_24_H_46_O_2_	[M‐H]^−^	366.3492	−1.6	<0.1
23	Tricosanoic acid (23:0)	15.6	C_23_H_46_O_2_	[M‐H]^−^	354.3493	−1.3	<0.1
24	Lignoceric acid (24:0)	16.8	C_24_H_48_O_2_	[M‐H]^−^	368.3647	−1.9	0.1
25	Pentacosanoic acid (25:0)	18.0	C_25_H_50_O_2_	[M‐H]^−^	382.3806	−1.2	0.1
26	Cerotic acid (26:0)	19.1	C_26_H_52_O_2_	[M‐H]^−^	396.3961	−1.6	0.1
27	Heptacosanoic acid (27:0)	19.8	C_27_H_54_O_2_	[M‐H]^−^	410.4118	−1.5	0.1
28	Montanic acid (28:0)	21.1	C_28_H_56_O_2_	[M‐H]^−^	424.4277	−0.8	<0.1

^a^ Please refer to Figure 2 for the peak numbering.

The related retention time (*tR*), formula, accurate mass (*u*), Δ mass error (ppm), and relative abundance are provided.

Relative abundance, based on areas as provided by Compound Discoverer, was used for a quantitative comparison of the tentatively identified FAs. The most abundant FAs were unsaturated ones, with a percentage of 72% and with a prevalence of carbon chain lengths of C16 and C18 (Figure 2B and D). Two MUFAs and three PUFAs were the most abundant species, with oleic and palmitoleic acid present at 10.3 and 6.2%, respectively. Linoleic, linolenic, and hexadecadienoic acids were in the percentage of 21.5, 21.3, and 10.3%, respectively. A smaller percentage of SFAs (28%) was also present in the microalgae sample, consisting primarily of C16 carbon chain length (Figure 2C). The most abundant acid was palmitic acid (23.0%), with a small proportion of stearic acid (2.2%). The qualitative identification highlights that *C. vulgaris* species could serve both as a good nutrition source when incorporated into diets, and a potential candidate for biodiesel production, since the amount and ratio of saturated and unsaturated FAs is fundamental to whether microalgae can be used as a biofuel feedstock. Our data are entirely according to the literature [36, 37, 38, 39]. Furthermore, the qualitative identification confirmed that the use of sulfuric acid was not only adequate for the break of the cell walls but also it was helpful for the complete hydrolysis of all lipids present in the microalgae.

### 3.3 Method validation

Even though untargeted analysis is a powerful approach for the qualitative identification of complex mixtures, absolute quantification is necessary to obtain more reliable data and share the obtained results more straightforwardly from one laboratory to another. To date, the most widely employed hyphenated technique for the profiling and the absolute quantification of FAs is GC‐MS, which can be highly laborious since it requires chemical derivatization due to the nonvolatile nature and low thermostability of these compounds. In the last years, LC‐MS/MS platforms have taken root in FA profiling, especially for their suitability in analyzing both polar and nonpolar, nonvolatile, or thermolabile compounds [40]. Usually, quantitative analysis is carried out by low‐resolution MS based on multiple reaction monitoring (MRM) acquisition. The MRM acquisition disadvantages are linked to the limited number of targets and low‐resolution MS generally employed. Since FAs do not have a fragmentation pattern, HRMS was necessary for their identification to compensate for the lack of characteristic product ions at the MS/MS level. The fragmentation patterns of FAs, in the negative‐ion mode, are primarily represented by the loss of a water molecule from saturated and some of the MUFAs species, the loss of carbon dioxide from the majority of unsaturated FA species, and the loss of acetate group. The intensity of these characteristic ion peaks depends on FAs species structures and collision conditions [41].

DDA was employed for the untargeted profiling and absolute quantification of the 28 tentatively identified FAs.

#### 3.3.1 Selectivity, precision, and recovery

Standard compounds were used to compare retention times and MS/MS spectra. The nonspiked extract was used to assess the absence of interference at the analyte retention times. However, endogenous amounts of the analytes were detected. Initially, recoveries were calculated for the 28 FAs subtracting the endogenous amount (*C*_0_), following Eq. 2. The proposed method accuracy was expressed as average *R*% at three spiking levels (*C*_1_, *C*_2_, *C*_3_) with related RSD (Table 2). Satisfactory assay accuracy ranging from 78 to 100% was achieved.

**TABLE 2:** Extraction recoveries (*R*, %) of 28 tentatively identified FAs at three different concentration levels (*C*_1_: 0.020 μg/mL; *C*_2_: 0.50 μg/mL; *C*_3_ 1.00 μg/mL)

	(*R* ± RSD)%		Precision (*C*_2_) (%)			
Fatty acid	*C*_1_	*C*_2_	*C*_3_	Intraday	Interday	*R* ^2^
Myristoleic acid (14:1)	(78 ± 2)%	(98 ± 1)%	(97 ± 1)%	10%	5%	0.9878
Myristic acid (14:0)	(96 ± 2)%	(99 ± 2)%	(98 ± 1)%	8%	3%	0.9865
Pentadecanoic acid (15:0)	(85 ± 4)%	(97 ± 3)%	(96 ± 2)%	3%	7%	0.9865
Hexadecadienoic acid (16:2)	(95 ± 3)%	(95 ± 1)%	(94 ± 1)%	8%	2%	0.9977
Palmitoleic acid (16:1)	(79 ± 3)%	(98 ± 2)%	(97 ± 3)%	5%	3%	0.9987
Palmitic acid (16:0)	(97 ± 3)%	(99 ± 2)%	(98 ± 2)%	5%	2%	0.9978
Heptadecenoic acid (17:1)	(85± 3)%	(97 ± 3)%	(95 ± 4)%	7%	4%	0.9867
Margaric acid (17:0)	(85 ± 3)%	(92 ± 3)%	(90 ± 2)%	5%	2%	0.9875
Linolenic acid (18:3)	(97 ± 2)%	(99 ± 1)%	(99 ± 2)%	7%	4%	0.9959
Linoleic acid (18:2)	(87 ± 2)%	(90 ± 2)%	(89 ± 3)%	6%	4%	0.9988
Oleic acid (18:1)	(98 ± 2)%	(100 ± 1)%	(99 ± 1)%	6%	3%	0.9998
Stearic acid (18:0)	(78 ± 4)%	(95 ± 4)%	(94 ± 3)%	6%	9%	0.9872
Nonadecenoic acid (19:1)	(80 ± 2)%	(98 ± 3)%	(97 ± 2)%	4%	2%	0.9863
Nonadecanoic acid (19:0)	(89 ± 3)%	(98 ± 4)%	(97 ± 3)%	7%	3%	0.9877
Eicosadienoic acid (20:2)	(84 ± 4)%	(97 ± 2)%	(96 ± 2)%	7%	4%	0.9854
Gadoleic acid (20:1)	(82 ± 2)%	(94 ± 3)%	(94 ± 2)%	6%	2%	0.9867
Arachidic acid (20:0)	(98 ± 2)%	(100 ± 1)%	(99 ± 1)%	6%	2%	0.9988
Heneicosenoic acid (21:1)	(85 ± 2)%	(97 ± 3)%	(96 ± 2)%	8%	5%	0.9887
Heneicosanoic acid (21:0)	(82 ± 3)%	(89 ± 3)%	(90 ± 1)%	6%	4%	0.9898
Brassidic acid (22:1)	(80 ± 4)%	(99 ± 1)%	(99 ± 2)%	7%	4%	0.9897
Behenic acid (22:0)	(88 ± 3)%	(90 ± 2)%	(89 ± 1)%	7%	3%	0.9879
Tricosanoic acid (23:0)	(84 ± 3)%	(97 ± 2)%	(96 ± 3)%	8%	4%	0.9984
Nervonic acid (24:1)	(85 ± 5)%	(95 ± 3)%	(94 ± 2)%	7%	2%	0.9972
Lignoceric acid (24:0)	(89 ± 2)%	(98 ± 3)%	(97 ± 3)%	8%	5%	0.9969
Pentacosanoic acid (25:0)	(98 ± 2)%	(99 ± 1)%	(98 ± 2)%	6%	5%	0.9978
Cerotic acid (26:0)	(87 ± 2)%	(95 ± 2)%	(94 ± 3)%	8%	5%	0.9888
Heptacosanoic acid (27:0)	(85 ± 2)%	(97 ± 3)%	(93 ± 2)%	7%	3%	0.9889
Montanic acid (28:0)	(84 ± 3)%	(95 ± 3)%	(95 ± 1)%	8%	5%	0.9899

Method reproducibility was determined by the intra‐ and interday precision calculation in extracts spiked with FAs at an intermediate fortification value (*C*_2_). Intraday accuracy was determined from five parallel experiments within 1 day, and intraday accuracy was determined from three parallel experiments for three consecutive days. The results showed that the intra‐ and interday RSDs were lower than 10 and 9%, respectively (Table 2), indicating that the method achieved satisfactory reproducibility and high performance.

#### 3.3.2 Calibration curve and matrix effect

Validation of the analytical method ideally requires calibration curves to be built in the same matrix, especially using ESI MS, because components in the sample can lead to ME, namely signal enhancement or suppression. In this work, the analytical validation was carried out on microalgae, and the background subtraction method was employed for building calibration curves. Absolute area values from the calibration curves were used to evaluate the ME (Eq. 3) and assess the impact of signal enhancement or suppression due to matrix components without using pure deuterated standards for each analyte. ME was not significant (97 ± 2%).

Good linearity was achieved for all analytes in the tested concentration range (0.25‐47.00 μg/mL), and the calibration curve equations were linear with correlation coefficients (*R* ^2^) ≥ 0.9854 (Table 2). The results indicated that this method was sensitive to the determination of free FAs in microalgae.

### 3.4 Absolute quantitation of fatty acids in *Chlorella vulgaris*

The validated method was finally applied to the quantitation of the 28 tentatively identified FAs (Table 3). Compared to the literature, this was the highest number of FAs quantified in one single analysis for microalgae samples. Few articles dedicated their attention to the absolute quantification of FAs in microalgae or similar organisms since only the qualitative percentage is usually provided [37, 38]. The only percentage analysis cannot establish the best strain for biodiesel production. Therefore, the quantitative analysis is fundamental to obtain critical decisions and select the microalgae with a high concentration of specific FAs.

**TABLE 3:** Results of FA quantitation in the *C. vulgaris*

Analyte	Concentration (mg/g) ± RSD
Palmitic acid (16:0)	135.01 ± 0.16
Linoleic acid (18:2)	86.31 ± 0.05
Linolenic acid (18:3)	85.37 ± 0.15
Hexadecadienoic acid (16:2)	54.16 ± 0.12
Oleic acid (18:1)	47.16 ± 0.06
Palmitoleic acid (16:1)	35.24 ± 0.02
Pentadecanoic acid (15:0)	30.08 ± 0.03
Stearic acid (18:0)	9.99 ± 0.04
Nonadecenoic acid (19:1)	5.00 ± 0.02
Gadoleic acid (20:1)	4.40 ± 0.02
Margaric acid (17:0)	4.12 ± 0.05
Lignoceric acid (24:0)	3.40 ± 0.02
Heptadecenoic acid (17:1)	2.88 ± 0.01
Cerotic acid (26:0)	2.76 ± 0.02
Pentacosanoic acid (25:0)	2.16 ± 0.02
Eicosadienoic acid (20:2)	1.80 ± 0.02
Behenic acid (22:0)	1.40 ± 0.01
Arachidic acid (20:0)	1.20 ± 0.02
Myristic acid (14:0)	0.84 ± 0.01
Heptacosanoic acid (27:0)	0.60 ± 0.01
Montanic acid (28:0)	0.31 ± 0.01
Nonadecanoic acid (19:0)	0.20 ± 0.01
Myristoleic acid (14:1)	0.12 ± 0.02
Tricosanoic acid (23:0)	0.11 ± 0.01
Brassidic acid (22:1)	0.09 ± 0.01
Heneicosanoic acid (21:0)	0.04 ± 0.01
Nervonic acid (24:1)	0.02 ± 0.01
Heneicosenoic acid (21:1)	<LOQ

Results are provided as mg of each FA compound per g of microalgae biomass (mean of six measurements).

It is difficult to determine an ideal FAs profile for biodiesel production; despite this, some general previsions based on cost production could be done. MUFAs, predominant in traditional high‐quality feedstocks, provide a reasonable balance between cold flow, oxidative stability, and combustion properties [42] and are preferable to SFAs or PUFAs. In our sample, oleic acid (18:1) and palmitoleic acid were present in a quantity of 47.16 mg/g and 35.25 mg/g, respectively. A small proportion of PUFAs in a biodiesel feedstock can benefit the biofuel flow properties, but the increasing levels could impact its oxidative stability [43]. As a result, production costs could increase to add antioxidant fuel additives. In this case, our results highlighted that linoleic, linolenic, and hexadecadienoic acids were in a high concentration (86.31, 85.37, and 54.16 mg/g, respectively).

The presence of SFAs improves the biodiesel combustion properties and gives rise to cold‐flow problems that limit its geographical market or year‐round suitability [44, 45]. The most abundant saturated species were palmitic and pentadecanoic acids with a concentration of 135.01 and 30.08 mg/g, respectively.

In addition to the overall FA profile, the partitioning of the FAs between different lipid classes is essential. Polar lipids primarily contain a high quantity of PUFAs, storage lipids are present in the form of TAGs, having a high content of SFAs and some unsaturated FAs and neutral lipids, for energy storage, consist of acylglycerols and free FAs, which have fatty acyl groups and a hydrogen atom attached to the glycerol backbone, respectively.

The high amount of omega‐3 and omega‐6 FAs make the analyzed *C. vulgaris* commercially useful for nutraceuticals and biofuel production.

A comparison with some recent articles was also provided. Schlotterbeck and his coworkers [20] determined, in an extract of *Undaria pinnatifida* alga, 421.2 ng/mL of hexadecatetranoic acid concentration. Due to its low abundance, the precursor ion quantification was carried out from the sequential window acquisition of all theoretical fragmentation mass spectra in data‐independent acquisition. Guan and coworkers [46] developed a GC‐MS method for simultaneous quantification of seven free FAs produced by wild‐type *Synechocystis* PCC 6803 cyanobacterium, its genetically engineered strain. The concentration ranged from a minimum of 16.4 μg/mL for arachidic acid to a maximum of 97.4 μg/mL for hexadecanoic acid. Linolenic, oleic, stearic, and linoleic acids were determined at 87.6, 45.1, 45.1, and 38.3 μg/mL, respectively. The absolute amount of C‐14:0, C‐16:0, and C‐18:0 by a validated GC‐MS/MS method was found to be 1.5‐1.7, 15.0‐42.5, and 4.2‐18.4 mg/g, respectively, in biodiesel obtained from six microalgal oils [47]. Kumari et al. [48] studied three different fresh macroalgal matrices (*Gracilaria corticata, Sargassum tennerrimum*, and *Ulva fasciata*) to find the best extraction method for FA quantification. Olmstead et al. [49] quantified three SFAs, four MUFAs, and two PUFAs in *Chlorella sp.*, obtained from the extraction and fractionation of neutral lipids, glycolipids, and phospholipids, and analyzed by GC‐MS studying the presence or deprivation of nitrogen during the growth. The transesterified FAs were reported as the sum of SFAs (25.5 and 41.9 mg/g), MUFA (8 and 26.1 mg/g), and PUFAs (67.0 and 58.6 mg/g) in *N* repleted or depleted growth condition, respectively.

## 4 CONCLUDING REMARKS

In our experimental condition, 28 FAs were quantified in the *C. vulgaris* extracts and palmitic, palmitoleic, oleic, linoleic, linolenic, and stearic acids were the most abundant ones. These specific FAs suggest that *C. vulgaris* is a good candidate for nutrition and biodiesel production. Further applicative studies should be carried out comparing different microalgal strains to improve biodiesel properties and to select the best microalgae or a combination of different microalgal strains with complementary FA profiles.

## AKNOWLEDGMENT

This work was supported by the Italian Ministry of University and Research (MUR, Italy) under grant PON for industrial research and experimental development ARS01_00881, ORIGAMI: Integrated biorefinery for the production of biodiesel from microalgae.

Additional funding was obtained by Sapienza University with a project: “Microalgae as a source of bioactive compounds: chromatographic fractionation characterization of peptides and lipids and their mass spectrometric identification” protocol n° RM11715C82118E74.

## CONFLICT OF INTEREST

The authors have declared no conflict of interest.

## Supporting information

TableS1‐S2

TableS3
