# Supplementary material for: Profiling and quantitative analysis of underivatized fatty acids in Chlorella vulgaris microalgae by liquid chromatography‐high resolution mass spectrometry
Source: J Sep Sci. 2021 Jun 24;44(16):3041–51. doi: 10.1002/jssc.202100306 (PMC8453725; doi:10.1002/jssc.202100306)
Supplement: Supplementary file 1 — TableS1‐S2 [file JSSC-44-3041-s001.docx]

**Profiling and quantitative analysis of underivatized fatty acids in Chlorella vulgaris microalgae by liquid chromatography-high resolution mass spectrometry**

Carmela Maria Montone^1^, Sara Elsa Aita^1^, Chiara Cavaliere*^1^, Martina Catani^2^, Andrea Cerrato^1^, Susy Piovesana^1^, Aldo Laganà^1,3^, Anna Laura Capriotti^1^

^1^ Department of Chemistry, Sapienza University of Rome, Piazzale Aldo Moro 5, 00185 Rome, Italy

^2^ Department of Chemistry and Pharmaceutical Sciences, University of Ferrara, Via L. Borsari 46, Ferrara, 44121, Italy

^3^CNR NANOTEC, Campus Ecotekne, University of Salento, Via Monteroni, 73100 Lecce, Italy

***Corresponding author**:

Chiara Cavaliere

Department of Chemistry, Università di Roma “La Sapienza”

Piazzale Aldo Moro 5

00185 Rome, Italy

E-mail: chiara.cavaliere@uniroma1.it

**Table S1**: List of 28 standard FAs and 1 standard TAG, 1 standard DAG, and five phospholipids with their abbreviation, CAS number and supplier.

| **Analyte** | **Abbreviation** | **CAS Number** | **Supplier** |
| --- | --- | --- | --- |
| Myristoleic acid | 14:1 | 544-64-9 | Merck |
| Myristic acid | 14:0 | 544-63-8 | Merck |
| Pentadecanoic acid | 15:0 | 1002-84-2 | Merck |
| Hexadecadienoic acid | 16:2 | 5070-03-01 | Larodan |
| Palmitoleic acid | 16:1 | 373-49-9 | Merck |
| Palmitic acid | 16:0 | 57-10-3 | Merck |
| Heptadecenoic acid | 17:1 | 29743-97-3 | Merck |
| Margaric acid | 17:0 | 506-12-7 | Merck |
| Linolenic acid | 18:3 | 463-40-1 | Merck |
| Linoleic acid | 18:2 | 60-33-3 | Merck |
| Oleic acid | 18:1 | 112-80-1 | Merck |
| Stearic acid | 18:0 | 57-11-4 | Merck |
| Nonadecenoic acid | 19:1 | 73033-09-7 | Merck |
| Nonadecanoic acid | 19:0 | 646-30-0 | Merck |
| Eicosadienoic acid | 20:2 | 2091-39-6 | Merck |
| Gadoleic acid | 20:1 | 29204-02-02 | Larodan |
| Arachidic acid | 20:0 | 506-30-9 | Merck |
| Heneicosenoic acid | 21:1 | 2363-71-5 | Larodan |
| Heneicosanoic acid | 21:0 | 3515-84-2 | Merck |
| Brassidic acid | 22:1 | 506-33-2 | Larodan |
| Behenic acid | 22:0 | 112-85-6 | Merck |
| Tricosanoic acid | 23:3 | 2433-96-7 | Merck |
| Nervonic acid | 24:1 | 506-37-6 | Merck |
| Lignoceric acid | 24:0 | 557-59-5 | Merck |
| Pentacosanoic acid | 25:0 | 506-38-7 | Larodan |
| Cerotic acid | 26:0 | 506-46-7 | Merck |
| Heptacosanoic acid | 27:0 | 7138-40-1 | Merck |
| Montanic acid | 28:0 | 506-48-9 | Merck |
| 1,3-dipentadecanoyl-2-oleoyl-glycerol | 15:0-18:1-15:0 TAG | 869990-13-6 | Merck |
| 1-Palmitoleoyl-2-pentadecanoyl-sn-glycerol | 15:0-18:1 DAG | 67889-40-1 | Merck |
| 1,2-dimyristoyl-sn-glycero-3-phosphate | 14:0 PA | 80724-31-8 | Merck |
| 1,2-dimyristoyl-sn-glycero-3-phospho-(1'-rac-glycerol) | 14:0 PG | 200880-40-6 | Merck |
| 1,2-dimyristoyl-sn-glycero-3-phosphocholine | 14:0 PC | 18194-24-6 | Merck |
| 1,2-dimyristoyl-sn-glycero-3-phosphoethanolamine | 14:0 PE | 998-07-2 | Merck |
| 1,2-dimyristoyl-sn-glycero-3-phospho-L-serine | 14:0 PS | 105405-50-3 | Merck |

**Table S2**: Hydrolysis percentages for the seven standard lipids employed for the optimization, at every tested temperature and reaction time.

|  | **25°C** | | | **70°C°C** | | | **140°C** | | |
| --- | --- | --- | --- | --- | --- | --- | --- | --- | --- |
|  | **60 min** | **90 min** | **Overnight** | **60 min** | **90 min** | **Overnight** | **60 min** | **90 min** | **Overnight** |
| 15:0-18:1-15:0 TG | (10±1)% | (20±2)% | (25±2)% | (60±3)% | (80±4)% | (78±4)% | (78±4)% | (100±5)% | (98±5)% |
| 15:0-18:1 DAG | (20±2)% | (25±2)% | (25±2)% | (50±3)% | (82±4)% | (80±4)% | (85±4)% | (100±5)% | (97±5)% |
| 14:0 PA | (11±1)% | (22±2)% | (24±2)% | (40±3)% | (65±4)% | (75±4)% | (60±4)% | (98±5)% | (99±5)% |
| 14:0 PG | (12±1)% | (24±2)% | (25±2)% | (38±3)% | (65±4)% | (75±4)% | (65±4)% | (99±5)% | (98±5)% |
| 14:0 PC | (14±1)% | (26±2)% | (26±2)% | (40±3)% | (64±4)% | (75±4)% | (68±4)% | (98±5)% | (99±5)% |
| 14:0 PE | (15±1)% | (26±2)% | (26±2)% | (40±3)% | (67±4)% | (78±4)% | (67±4)% | (97±5)% | (98±5)% |
| 14:0 PS | (15±1)% | (26±2)% | (10±2)% | (42±3)% | (68±4)% | (77±4)% | (69±4)% | (99±5)% | (98±5)% |
